# SGLT2 inhibition ameliorates nano plastics-induced premature endothelial senescence and dysfunction

**DOI:** 10.1038/s41598-023-33086-2

**Published:** 2023-04-17

**Authors:** Bikalpa Dhakal, Saugat Shiwakoti, Eun-Young Park, Ki-Woon Kang, Valérie B. Schini-Kerth, Sun-Hwa Park, Hye-Young Ji, Joon Seok Park, Ju-Young Ko, Min-Ho Oak

**Affiliations:** 1 College of Pharmacy, Mokpo National University, 1666 Yeongsan-ro, Cheonggye-Myeonn, Muan-Gun, Jeonnam 58554 Republic of Korea; 2 Division of Cardiology, College of Medicine, Heart Reasearch Institute and Biomedical Research Institute, Chung-Ang University Hospital, Chung-Ang University, Seoul, 06974 Republic of Korea; 3 Regenerative Nanomedicine, Faculty of Pharmacy, UMR 1260 INSERM (French National Institute of Health and Medical Research), University of Strasbourg, 67000 Strasbourg, France; 4 Life Science Institute, Daewoong Pharmaceutical, Yongin, Gyeonggido 17028 Republic of Korea

**Keywords:** Cardiovascular biology, Vascular diseases, Molecular medicine

## Abstract

Nano plastics (NPs) have been a significant threat to human health and are known to cause premature endothelial senescence. Endothelial senescence is considered one of the primary risk factors contributing to numerous cardiovascular disorders. Recent studies have suggested that inhibition of sodium glucose co-transporter (SGLT2) ameliorates endothelial senescence and dysfunction. Therefore, our study intends to explore the role of SGLT2 in NPs-induced endothelial senescence and dysfunction. Porcine coronary artery and its endothelial cells were treated with NPs in the presence or absence of Enavogliflozin (ENA), a SGLT2 inhibitor and then SGLTs expression, senescence markers and vascular function were evaluated. NPs significantly up-regulated SGLT2 and ENA significantly decreased NPs-induced senescence-associated-*β*‐gal activity, cell‐cycle arrest, and senescence markers p53 and p21 suggesting that inhibition of SGLT2 prevents NPs-induced endothelial senescence. In addition, ENA decreased the formation of reactive oxygen species with the downregulation of Nox2, and p22^phox^. Furthermore, SGLT2 inhibition also up regulated the endothelial nitric oxide synthase expression along with improving vascular function. In conclusion, premature endothelial senescence by NPs is, at least in part, associated with SGLT2 and it could be a potential therapeutic target for preventing and/or treating environmental pollutants-induced cardiovascular disorders mediated by endothelial senescence and dysfunction.

## Introduction

Nano plastics (NPs) are plastic particles of a size range less than 0.1 µm that are either deliberately incorporated in various industrial products or may be formed by the fragmentation and degradation of large-size plastics^1^. NPs are widespread in the land field, water as well as in air and because of their small size, they permeate through various biological barriers causing multiple health hazards^2^. Some studies have shown that NPs exposure is mainly via ingestion, inhalation, or dermal contact and is linked with numerous cardiovascular, pulmonary, metabolic & neuronal complications^3^. NPs induce premature endothelial senescence and dysfunction which is accompanied by an increase in intracellular oxidative stress^4^. Endothelial cells (ECs) senescence is characterized by irreversible cell cycle arrest, inflammation, oxidative stress, and downregulation of endothelial nitric oxide synthase (eNOS)-derived nitric oxide (NO) formation. ECs senescence can be induced by various factors like DNA damage, pollutants, high glucose, angiotensin II, and radiation, which promotes endothelial dysfunction and is a significant risk factor for the development of cardiovascular disorders (CVDs)^4–6^.

Studies done in various experimental models suggest that NPs disrupt glucose metabolism, alter energy utilization and lead to metabolic disorders^7,8^. Recently, glucose metabolism is becoming a major subject of matter linked with premature and replicative endothelial senescence in a particular concern with sodium-glucose co-transporter type 2 (SGLT2). Inhibition of SGLT2 has been shown to downregulate the formation of reactive oxygen species (ROS), which is one of the major inducers of endothelial senescence and dysfunction^9,10^. Also, SGLT2 inhibitors show effectiveness to restore endothelial homeostasis via decreasing endothelial senescence induced by multiple stressors, improving vascular reactivity, suppressing intracellular oxidative stress, and balancing the local angiotensin system^10,11^. Moreover, several scientific evidence suggests that SGLT2 inhibitors improve energy metabolism, autophagy, lysosomal degradation, and vascular function, and decrease the inflammatory response, which has established it as a potential agent for the prevention of vascular ageing as well as vascular ageing-related disorders^12,13^. Since inhibition of SGLT2 has protective cardiovascular effects^9,14^ it can be a potential target to prevent the initiation of CVDs caused by various possible contaminants, which yet remain unexplored.

Standing on the fact that NPs are potent risk factors for cardiometabolic disorders and SGLT2 inhibitors are suitable candidates against cardiovascular complications, investigations were carried out to find the relation between NPs-induced endothelial senescence and SGLT2. Furthermore, this study intends to explore whether SGLT2 inhibition could improve NPs-induced premature ECs senescence and vascular dysfunction. NPs exposure significantly up-regulated SGLT2 expression and senescence-associated-*β*‐galactosidase (SA‐*β*‐gal) activity, one of the prominent senescence markers whereas inhibition of SGLT2 significantly reduced SA‐β‐gal activity in PCA and PCAECs. Inhibition of SGLT2 also prevented the NPs-induced upregulation of senescence (p53, p21), and NADPH oxidase (Nox2 and p22^phox^) markers and inducers of redox-sensitive endothelial senescence. In addition, SGLT2 inhibition improved NPs‐induced vascular dysfunction along with the substantial up-regulation of eNOS.

## Results

### Inhibition of SGLT2 prevents NPs-induced premature Endothelial Senescence in PCA and PCAECs

The physicochemical characteristics of NPs were determined initially using Field emission scanning electron microscopy (FE-SEM) and dynamic light scattering instrument (DLS). FE-SEM image demonstrated that the morphology of the NPs was spherical and like those specified by the manufacturer (Fig. S1a). DLS results showed that the zeta potential and the hydrodynamic diameter of NPs were respectively found to be − 58.8 ± 0.39 mV and 22.41 ± 0.63 nm, indicating that NPs were not aggregated in solution (Fig. S1b,c).

**Figure 1:**
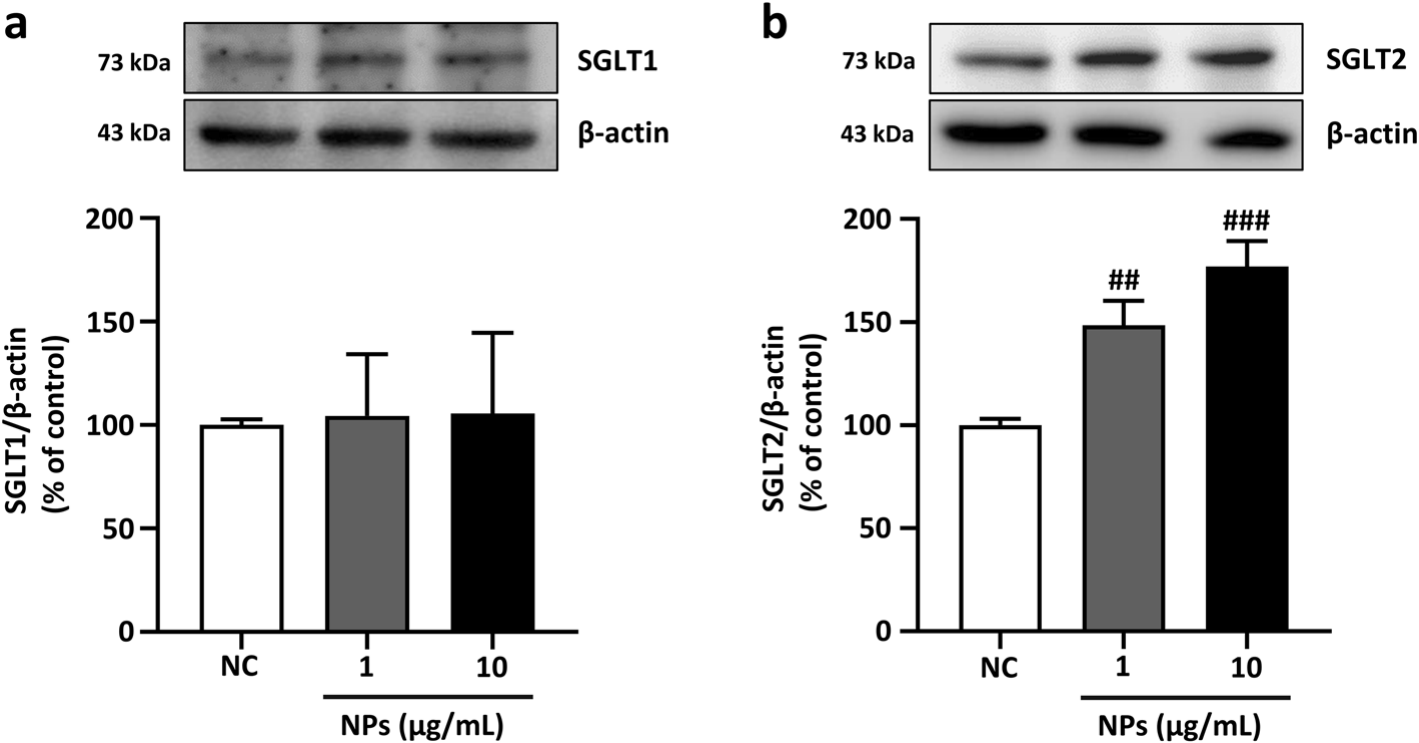
NPs exposure increases SGLT2 expression in PCAECs. PCAECs were incubated in the presence of different NPs concentrations (1 and 10 μg/mL) for 24 h and level of SGLT1 and SGLT2 expression were determined by western blot. (**a–b**) Representative immunoblots of SGLT1 and SGLT2 with their respective cumulative data. Data are the mean ± SEM (n = 3–5). ##*p* < 0.01, ###*p* < 0.001 versus NC: negative control (complete media).

**Figure 2:**
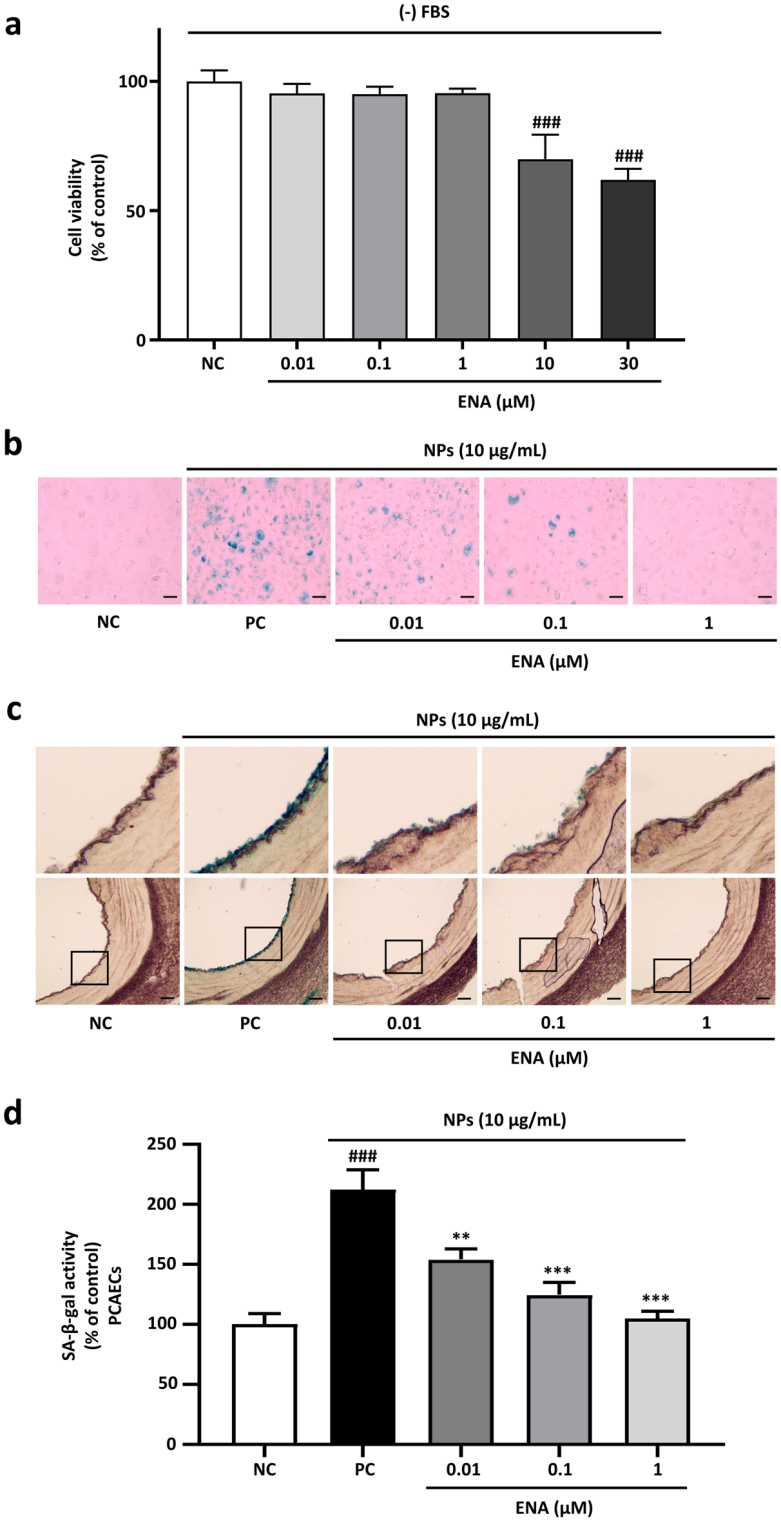
Inhibition of SGLT2 suppresses NPs-induced premature senescence in cultured and native ECs. (**a**) Cell viability of PCAECs with different doses of ENA. Data are the mean ± SEM (n = 6). ###*p* < 0.001 versus NC (complete media). (**b–c**) SA-*β*-gal activity was determined by X gal staining in PCA and PCAECs treated with or without NPs (10 μg/mL) alone or with ENA (0.01, 0.1, 1 μM) for 24 h. Representative staining images of PCAECs showing SA-*β*-gal (blue stain), and PCA rings, respectively; scale bar = 100 μM. (**d**) Cumulative SA‐*β*‐gal activity in PCAECs as a percentage of control. Results are expressed as mean ± SEM (n = 3–4). **p < 0.01; ***p < 0.001 versus PC: positive control (complete media + NPs 10 μg/mL), ###*p* < 0.001 vs. NC (complete media).

The effect of NP exposure on the expression of SGLT1 and SGLT2 was assessed using western blot analysis by incubating PCAECs with different concentrations of NPs (1, 10 μg/mL) for 24 h. The treatment dose and duration of NPs was selected referred to our previous study, where NPs at 1 and 10 µg/mL for 24 h could significantly induce endothelial senescence and dysfunction^4^. The expression of SGLT2, not SGLT1, was significantly increased by NPs with increasing concentrations (Fig. 1a,b). A further investigation was conducted to determine how SGLT2-inhibition affects NPs-induced endothelial senescence by using ENA as a representative SGLT2 inhibitor. Different concentrations of ENA (0.01, 0.1, 1, 10, and 30 μM) treated PCAECs were tested for their viability till 24 h. Results showed that the dose above 1 μM showed significant toxicity resulting in decrease cell viability up to 69.8% and 61.2% in 10 and 30 μM concentration respectively (Fig. 2a). Thus, further experiments were carried out using 1 μM as the highest testing concentration of ENA.

To further explore the effect of SGLT2 inhibition on NPs exposure, the downstream experiments were performed with stronger dose of NPs (10 µg/mL) which is entirely intended to be sure of the response of SGLT2 inhibition by ENA at different concentration against maximum NPs exposure. SA‐*β*‐gal activity was determined in PCA and PCAECs by incubating them with NPs (10 µg/mL) alone or in the presence of different doses of ENA (0.01, 0.1, 1 µM) followed by cytochemical detection with x-gal reagent. NPs exposure increased the SA‐*β*‐gal activity in PCA and PCAECs in a dose-dependent manner as observed by increase in the number of SA‐*β*‐gal positive cells. However, this increase in SA‐*β*‐gal activity by NPs exposure was significantly reduced by inhibiting SGLT2 in a concentration-dependent manner (Fig. 2b–d).

**Figure 3:**
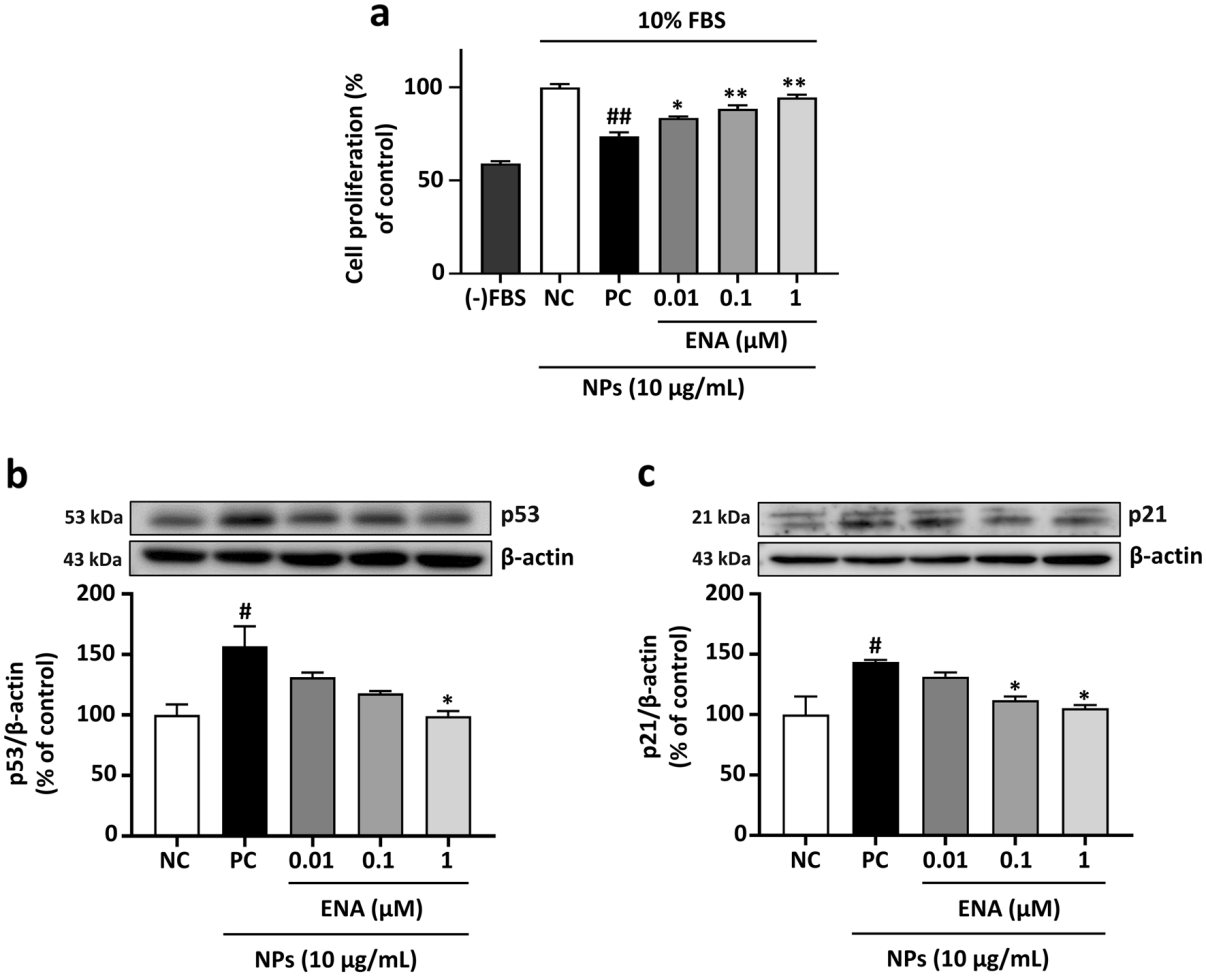
SGLT2 inhibition increases cell proliferation and down-regulates the cell cycle regulatory proteins expression altered by NPs exposure. (**a**) Cell proliferation of PCAECs treated with or without NPs (10 μg/mL) alone or with ENA (0.01, 0.1, 1 μM) for 24 h. Data are the mean ± SEM (n = 6). *p < 0.05; **p < 0.01 vs. PC (complete media + NPs 10 μg/mL); ##*p* < 0.01 vs. NC (complete media). (**b-c**) Representative immunoblots for cell cycle regulatory proteins: p53 and p21 (lower band) with their corresponding cumulative data. Data are the mean ± SEM (n = 3–4). *p < 0.05 versus PC; #*p* < 0.05 versus NC.

NPs-induced endothelial senescence is associated with decreased cell proliferation^4^. Therefore, the effect of SGLT2 inhibition on cell proliferation in NPs-induced endothelial senescence was studied. NPs exposure significantly reduced cell proliferation of PCAECs and SGLT2 inhibition increased it in a concentration-dependent manner (Fig. 3a).

The study further examined the expression of cell cycle-regulatory proteins, like p53 and p21 proteins, prominent markers of senescence in endothelial cells. Consistent with SA‐*β*‐gal activity and cell proliferation, NPs exposure substantially upregulated the expression of senescence markers p53 and p21 whereas inhibition of SGLT2 prevented their expression significantly (Fig. 3b,c). Overall, these results suggest that inhibition of SGLT2 potentially reduced NPs-induced endothelial senescence with subsequent downregulation of p53 and p21.

### Enavogliflozin does not interfere with NPs uptake by PCAECs

**Figure 4:**
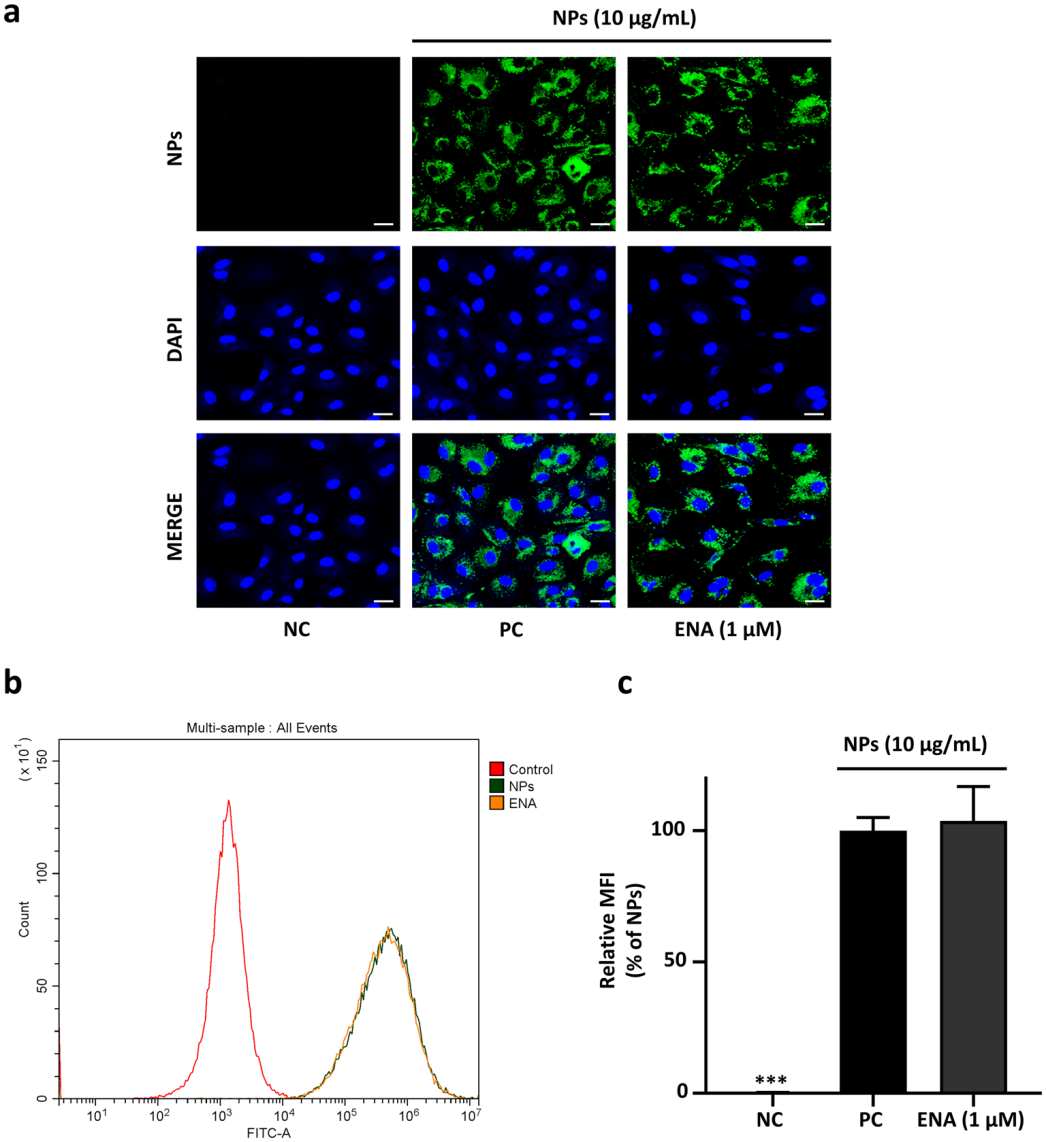
Enavogliflozin does not interfere with NPs uptake by PCAECs. (**a**) Representative confocal microscopy images of NPs internalization in PCAECs treated with or without NPs (10 μg/mL) alone and with ENA (1 μM) for 24 h. DAPI: diamidine-2-phenylindole dihydrochloride; MERGE: the merged image of NPs + DAPI; scale bar = 20 μM. (**b**) Representative histogram of NPs internalization analyzed with flow cytometry; (**c**) The mean of three independent experiments is presented as the relative mean fluorescence intensity (MFI). The values were normalized against the MFI of PC as a reference. The mean fluorescence intensity indicates the accumulation of NPs in cells. Data are the mean ± SEM (n = 3). ***p < 0.001 versus PC (complete media + NPs 10 μg/mL).

Confocal microscopy and flow cytometry were performed to investigate the effect of ENA on NPs uptake by ECs. To investigate the NPs internalization, cells were incubated for 24 h with or without NPs (10 µg/ml) alone or with ENA (1 µM). The internalization was analyzed by using a laser scanning confocal microscope after 24 h incubation and flow cytometry analyses were conducted to determine the efficiency of NPs uptake by PCAECs in the presence of ENA. The cytoplasmic accumulation of NPs was significant and uniform in both NPs and NPs with ENA-treated cells compared to non-treated cells (Fig. 4a) and intensity in both NPs and NPs with ENA treated cells were similar and substantially higher compared to non-treated cells (Fig. 4b,c) confirming that ENA did not affect NPs uptake. Overall, NPs were well internalized into PCAECs, and this effect was not affected by the ENA treatment which suggests that inhibition of NPs-induced endothelial senescence by ENA unlikely to be explained by the prevention of NPs uptake.

### SGLT2 inhibition reduces oxidative stress caused by NPs

**Figure 5:**
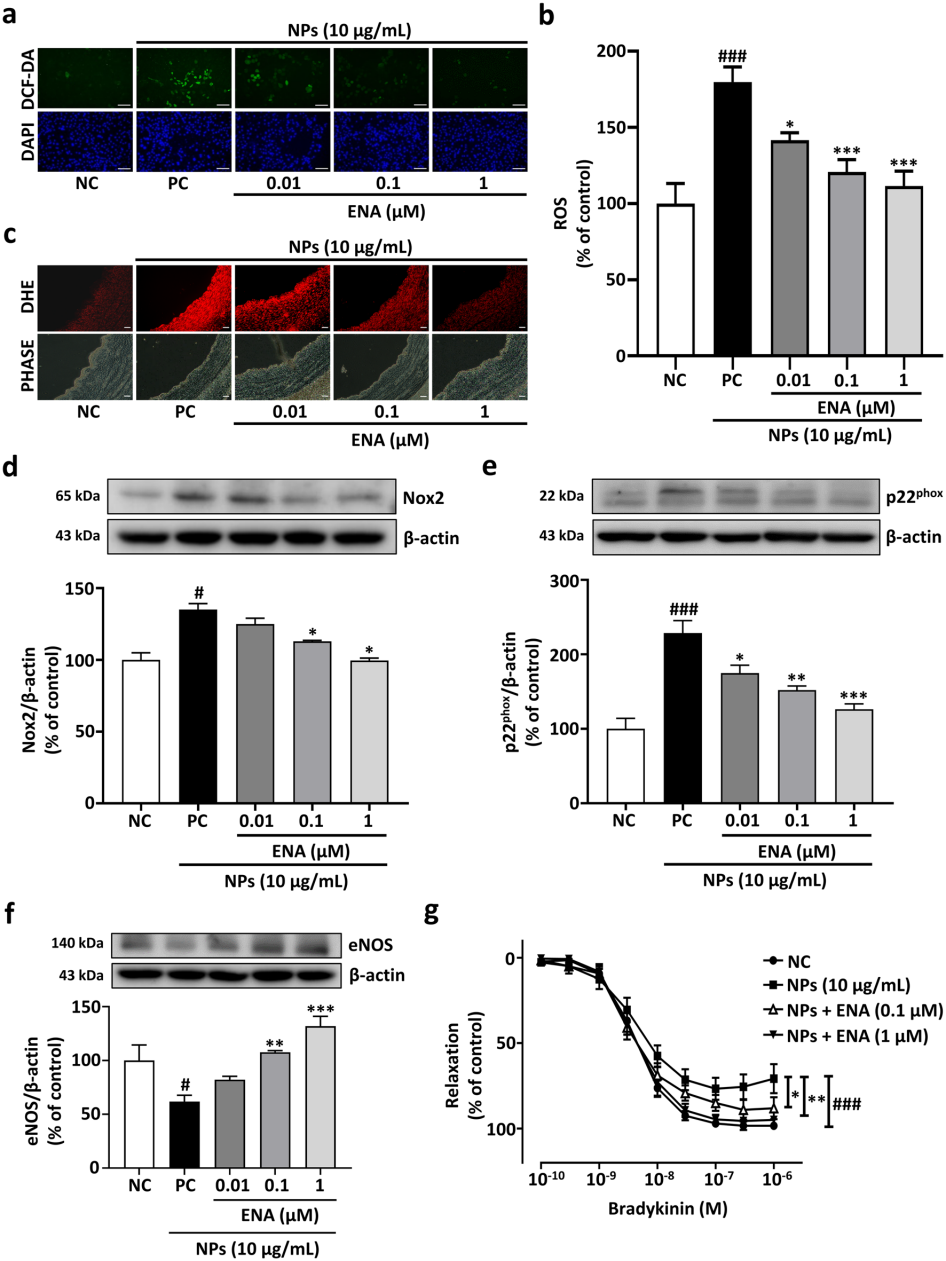
SGLT2 inhibition reduces NPs induced oxidative stress and prevents endothelial dysfunction. (**a**) Representative fluorescence microscopy images of DCF‐DA in live adherent ECs with corresponding 4′,6-Diamidino-2-phenylindole (DAPI) staining after the 24 h treatment of NPs (10 μg/mL) alone or with ENA (0.01, 0.1, 1 μM); scale bar = 500 μM. (**b**) Quantification of relative DCF-DA fluorescence intensity measured as a percentage of control. Data are the mean ± SEM (n = 5–6). *p < 0.05; ***p < 0.001 versus PC (complete media + NPs 10 μg/mL); ###*p* < 0.001 versus NC (complete media). (**c**) Representative microscopy images of ethidium fluorescence in arterial rings; scale bar = 100 μM. (**d-f**) Representative immunoblots of Nox2, p22^phox^ (upper band) and eNOS proteins with their corresponding cumulative data. Data are the mean ± SEM (n = 3). *p < 0.05; **p < 0.01; ***p < 0.001 versus PC; #*p* < 0.05; ###*p* < 0.001 vs. NC. (**g**) Concentration-relaxation curve in response to BK after 24 h treatment with NPs (10 μg/mL) alone or with ENA (0.1 and 1 μM). Data are the mean ± SEM (n = 7–13). *p < 0.05; **p < 0.01 versus PC; ###*p* < 0.001 versus NC.

Investigations were carried out to determine whether inhibition of SGLT2 suppresses oxidative stress caused by NP, using two different redox-sensitive fluorescent dyes DCF-DA and DHE. As shown in Fig. 5a,b, exposure of PCAECs to NPs increased the fluorescent signal of DCF-DA reflecting an increased formation of ROS, which was prevented in a concentration-dependent manner by ENA. Similar findings were also observed using the redox-sensitive probe DHE in PCA, indicating that inhibition of SGLT2 reduced the increased ROS generation in response to NPs (Fig. 5c).

Since NPs-induced oxidative stress involves NADPH oxidase, known to be a major source of ROS in the ECs^4,15^, further studies explored the role of SGLT2 inhibition on NADPH oxidase. Western blotting analysis indicated that Nox2 and p22^phox^, membrane catalytic subunits of NADPH oxidase, were significantly upregulated by NPs and that SGLT2 inhibition suppressed the response significantly in 0.1 and 1 µM concentrations (Fig. 5d,e).

### Inhibition of SGLT2 prevents NPs-induced vascular dysfunction

Downregulation of eNOS is the characteristic of ECs senescence leading to vascular dysfunction^16^. Since NADPH is an important substrate for eNOS biosynthesis, NPs-induced upregulation of NADPH oxidase may affect the eNOS level in endothelial cells^17^. Thus, we investigated whether inhibition of SGLT2 is able to prevent eNOS down-regulation and improve vascular function impaired by NPs. According to the result, inhibition of SGLT2 was associated with increased levels of eNOS in NPs-treated PCAECs (Fig. 5f). The induction of senescence in ECs and the decreased level of eNOS causes ECs to be unable to regulate vascular homeostasis, causing vascular dysfunction in the body^18^. Therefore, we investigated whether inhibition of SGLT2 prevents NPs-induced vascular dysfunction associated with endothelial senescence. PCA rings were treated with or without NPs alone and in the absence or presence of different concentrations of ENA before the evaluation of vascular reactivity. Findings indicate that NPs significantly decreased the concentration-dependent relaxations to bradykinin with maximal effect (Emax) of 69.98% ± 8.33% compared to control, Emax 100.38% ± 3.38%, whereas PCA rings treated with different concentrations of ENA (0.1, 1 µM) increased relaxation exhibiting Emax of 84.81% ± 11.05% and 96.1% ± 6.25% respectively (Fig. 5g). Based on the findings, SGLT2 inhibition prevented endothelial dysfunction caused by NPs in a concentration-dependent manner.

## Discussion

The threat of environmental pollutants, such as NPs, is emerging as a global health concern. There is an unmet need to characterize their deleterious potential and to assess their risks to human health. The major findings of this study demonstrate that SGLT2 inhibition prevents NPs-induced premature endothelial senescence and dysfunction, decreases the level of ROS, and upregulate the eNOS-derived NO formation thereby preventing vascular dysfunction.

Environmental pollutants such as particulate matter, and micro and nano plastics can alter glucose metabolism and are considered prime risk factors for cardiometabolic disorders^19,20^. A growing body of experimental evidence also demonstrates a link between NPs exposure and cardiovascular risk factors such as irregular blood flow, disturbed heart rate, myocardial fibrosis, and cardiometabolic disease phenotype^21^. SGLT2 inhibitors such as dapagliflozin, canagliflozin, empagliflozin, and ipragliflozin are a novel class of blood sugar-lowering agents which are known to have potential cardioprotective activity^22^. Moreover, several scientific investigations have shown the evidence of SGLT2 inhibition improving the condition of severe heart failure in different animal models as well as in human trials independent of the glycemic control^23,24^. The cardiovascular benefits of SGLT2 inhibitors in heart failure, thus, could also be achieved in glucose-independent manner by acting as an antioxidant restoring oxidant-antioxidant balance through several mechanisms including inhibition of eNOS uncoupling, downregulation of NADPH oxidase expression, and attenuation of overexpressed superoxide dismutase^25^. Additionally, SGLT2 inhibitors have been shown to reduce inflammation and oxidative stress, both of which can contribute to the development and cardiovascular disease^26^. Enavogliflozin (DWP16001) is one of the selective SGLT2 inhibitors under development by Daewoong Pharmaceutical Co., Ltd (Seoul, Korea) which has recently approved by MFDS of Korea (Ministry of Food and Drug Safety). ENA has a lesser half maximal inhibitory concentration (IC_50_) of SGLT2, suggesting that ENA has a higher affinity for SGLT2^27^. In this study, NPs-induced premature endothelial senescence was significantly reduced by ENA treatment suggesting that SGLT2 inhibition could ameliorate NPs-induced premature senescence. In addition, treatment with NPs also significantly upregulated the expression of SGLT2 while no substantial change was seen in the expression of SGLT1. This finding further supports the notion that SGLT2 is associated with NPs-induced endothelial senescence and dysfunction.

The well-known pathway governing the cell cycle arrest in cellular senescence is the p53/p21 pathway which has the key function for the positive regulation of the cell cycle exit^28,29^. Various studies have shown that SGLT2 inhibitors downregulated the expression of p53 and p21, markers of cellular senescence, thereby suppressing premature senescence in different experimental models^9,30,31^. To further confirm the protective effect of SGLT2 inhibition on NPs-induced ECs senescence, we assessed the effect of ENA on senescence marker-associated pathways. In this study, treatment with NPs increased the expression level of senescence markers, p53 and p21, while treatment with ENA significantly reduced the expression of these senescence markers, supporting a protective role for SGLT2 inhibition towards NPs-induced senescence. In addition, ENA also reversed the NPs-induced loss of proliferative capacity in ECs. These findings demonstrated that inhibition of SGLT2 could prevent premature ECs senescence caused by NPs exposure.

Pollutants like polystyrene nanoparticles and fine dust can directly induce oxidative stress in ECs leading to premature ECs senescence and vascular dysfunction^4,32^. Most of the detrimental effects associated with NPs exposure are attributed to their ability to generate ROS and increase oxidative stress within cells^33,34^. Thus, reducing oxidative stress could be a major strategy for preventing NPs-induced cardiovascular complications. Recent studies have shown that premature endothelial senescence caused by the increased intracellular oxidative stress is associated with the upregulation of SGLT2 and its inhibition has been shown to suppress an increased level of ROS ultimately alleviating premature senescence^30,35^. The SGLT2 inhibitors have emerged as a potential agent against oxidative damage in tissues, not only due to their glucose-lowering properties but also by reducing free radical generation or by enhancing antioxidant action^36^. Similarly, in this study, the increased level of ROS due to NPs exposure was significantly decreased in PCAECs and PCA. These findings suggest that SGLT2 inhibition suppresses NPs-induced oxidative stress and SGLT2 inhibition could positively impact and delay the onset of NPs-induced premature senescence.

ROS derived from NADPH oxidase is considered to be the major regulator of ECs senescence and endothelial dysfunction contributing to atherosclerosis, diabetes, hypertension and other cardiovascular complications^37,38^. Moreover, exposure to environmental pollutants such as NPs has been shown to induce premature ECs senescence through NADPH oxidative-dependent stress^4^. Therefore, the expression level of NADPH oxidase subunits was assessed to determine the role of SGLT2 inhibition in NPs-induced NADPH oxidase-mediated oxidative stress. The increased expression of Nox2 and p22^phox^ by NPs exposure was significantly prevented by inhibition of SGLT2, supporting previous studies indicating that various SGLT2 inhibitors reduced the expression of NADPH oxidase and prevented NADPH-dependent oxidative stress in several animal models^39,40^. These results suggest that SGLT2 inhibition decreases NPs-induced endothelial oxidative stress, at least in part, by inhibiting the expression of NADPH oxidase, a major source of ROS in ECs.

Impaired endothelial function is the characteristic feature of ECs and is regarded as an indicator of future CVDs^41^. Premature ECs senescence induced by environmental pollutants is associated with increased intracellular oxidative stress causing endothelial dysfunction^4,32^. The occurrence of endothelial dysfunction is most likely due to the downregulation of eNOS with a subsequently reduced NO bioavailability^42^. Previous evidence has shown that SGLT2 inhibitors prevent the down-regulation of eNOS thereby improving the endothelial function in response to multiple stressors^30,43^. In this study, we observed that vascular dysfunction caused by the NPs exposure was significantly prevented by SGLT2 inhibition along with a significant increase in the eNOS level. This suggests that SGLT2 inhibition can prevent NPs-induced endothelial dysfunction, at least in part, by preventing the down-regulation of eNOS expression and by stimulating eNOS enzymatic activity. Numerous studies have shown that NO can be degraded directly in the presence of ROS and that this phenomenon not only reduces NO bioavailability but also produces deleterious superoxide anions O_2_^-^ which can induce oxidative damage, further promoting endothelial dysfunction^18,44,45^. Since SGLT2 inhibitors reduced ROS formation in endothelial cells^46^, such an effect may also prevent the oxidative degradation of NO, thus alleviating NPs-induced NADPH-dependent oxidative stress, ECs senescence and endothelial dysfunction. Thus, SGLT2 inhibition can be a potential target for the prevention of pollutants-induced vascular dysfunction.

The present study is based on in vitro*,* and ex vivo experiments but to extrapolate the findings to in vivo studies, there must be an accurate assessment of different types of plastics, their exposure routes, exposure concentration, and characterization of their accumulation in the human body. At present, it is challenging to accurately assess the accumulation and potential health effects of NPs in vivo due to complexity and unique properties of NPs^47,48^. Although there are few animal models, such as rodents and zebra fish, it is important to note that animal models have limitations and differences from humans^49,50^. Further research is needed to understand the effects of NPs on cardiovascular health, and compelling evidence is required on the quantification of its effects. Similarly, the mechanisms underlying the effect of NPs on energy intake and the cellular status after SGLT2 inhibition needs detailed understanding. Also, this may open the platform for further research on the detrimental effect of NPs exposure in cardiovascular health and its correlation with SGLT2.

## Conclusion

There is an emerging threat of environmental pollutants, such as NPs, as a major risk factor for CVDs, yet they are poorly explored. The present findings pinpoint a novel mechanism associated with NPs-induced deleterious impact on cardiovascular health and suggest that SGLT2 may be a potential contributor to the NPs-induced premature endothelial senescence and dysfunction and that SGLT2 inhibition could be a novel approach to combat premature vascular ageing and dysfunction caused by NPs. The observed protective effects of SGLT2 inhibition on NPs exposure were associated with the suppression of cell cycle regulatory proteins, inhibition of NADPH oxidase-mediated ROS formation, and increased eNOS-derived NO formation. This study provides preliminary data for use in further investigations of the relationships between NPs exposure and the role of SGLT2 in it. Overall, the inhibition of SGLT2 may be an interesting novel target against environmental pollutants such as NPs-induced CVDs mediated by premature endothelial senescence and dysfunction.

## Materials and methods

### Materials

Enavogliflozin (DWP16001) was obtained from Daewoong Pharmaceutical Co., Ltd (Seoul, Korea) in pure powder form and was dissolved in 10% DMSO in 1X Krebs solution, aliquoted and stored at 4 °C. All other chemicals and reagents were of analytical grade.

NPs were purchased from Bangs Laboratories,Inc and were mainly composed of polystyrene. The characterization of NPs was done using FE-SEM (Regulus 8230; Hitachi, Japan) and DLS instrument (Zetasizer Nano ZS90; Malvern Instruments, UK). The particle concentration was 10% solid (100 mg/mL) which were diluted in 10% dimethyl sulfoxide (DMSO) in phosphate buffer saline (PBS) to get a stock solution of 10 mg/mL. The stock solution (10 mg/mL) of NPs were diluted further in the culture medium to prepare the working concentration.

### PCAECs isolation and culture

Pig hearts from a local slaughterhouse (Mokpo, South Korea) were brought to the laboratory immediately after sacrifice and maintained at 4 °C in Krebs bicarbonate solution (concentration in mmol/L: KCl 4.7, NaCl 119, KH_2_PO_4_ 1.18, CaCl_2_ 1.25_,_ MgSO_4_ 1.18, D‐glucose 11 and NaHCO_3_ 1.25; pH 7.4). The investigation included the use of tissues obtained postmortem from a local slaughterhouse (Mokpo, South Korea) which adheres to the US Department of Agriculture’s Animal Cruelty and Slaughter Act guidelines, so the need for approval from the institutional animal ethics committee was waived (Mokpo, South Korea) following the US guidelines. The left anterior descending coronary artery was dissected soon after the arrival of the heart and cleared of loose connective tissues in Krebs bicarbonate solution. PCAECs were then isolated using 1 mg/ml of collagenase type I (Worthington) for 20 min at 37 °C and then cultured in T-flasks containing DMEM (Gen depot, USA) provided with 10% fetal bovine serum (FBS), penicillin (100 U/mL), streptomycin (100 U/mL) and fungizone (250 µg/mL) until confluence as described previously^51^. Since the NPs uptake was significantly hindered by serum^4,52^, cells were treated with NPs in serum free medium for 6 h followed by an 18 h incubation with 10% FBS medium. Premature senescence was induced in cells with NPs (10 µg/mL) and the role of SGLT2 was investigated using an SGLT2 inhibitor, ENA which was studied at different concentrations (0.01, 0.1, and 1 µM) in either the absence or presence of NPs and the untreated cells served as a control group.

### Senescence-associated *β*-galactosidase (SA-*β*-Gal) activity detection

Senescence-associated *β*-galactosidase (SA-*β*-Gal) activity is the characteristic feature of ageing cells and a histochemical marker for senescence^16^, which was detected by using the x-gal staining reagent (40 mM citric acid/Na phosphate buffer, 5 mM potassium ferrocyanide, 5 mM potassium ferricyanide, 2 mM MgCl_2,_ 150 mM NaCl, and 1 mg/mL X-gal in distilled water) with a slight adjustment of the method detailed previously^53^. Briefly, PCAECs were treated with or without NPs (10 µg/mL) alone and with ENA (0.01, 0.1, 1 µM) then incubated for 24 h. After that, cells were fixed with 4% paraformaldehyde, treated with x-gal staining reagent, and left overnight in a humidifying chamber at 37 °C. Finally, the cells were washed with 1X PBS and observed under a bright field microscope. The percentage of cells positively expressing SA-*β*-Gal activity was estimated by counting the number of blue-stained cells within the total cell population.

Similarly, PCA were cut into small rings of approximately 2–3 mm and treated with or without NPs (10 µg/mL) alone or with ENA (0.01, 0.1, 1 µM) then incubated for 24 h. Thereafter, PCA rings were incubated overnight with x-gal reagent after fixing with 4% paraformaldehyde. PCA rings were then submerged in optimum cutting temperature (OCT) solution (Leica Biosystems) and were sliced into 10 µm sections using a cryotome at − 25 °C. After all, the tissue slides were prepared and examined under a bright‐field microscope.

### Cell proliferation and cell viability assay

PCAECs were treated with or without NPs alone in the absence or presence of ENA (0.01, 0.1, 1 µM) after seeding in a 96‐well plate (1 × 10^4^ cells/well). CellTiter 96 Aqueous One Solution Cell Proliferation Assay (Promega Corporation, USA) was used to access the cell proliferation where quantification of proliferative response was done by using MTS tetrazolium solution (20 µL/well). Following incubation of 2–3 h, absorbance was measured at 490 nm in Enspire Multilabel Reader (Perkin Elmer Ltd.). PCAECs were incubated with different concentrations of ENA in a media without serum for 24 h to determine the effect of ENA on cell viability. After the incubation, cells were treated with (20 µL/well) of MTS tetrazolium solution like the cell proliferation assay and proceeded to determine the absorbance at 490 nm after 2–3 h in Enspire Multilabel Reader. Different individual assays were carried out in triplicate. Data were expressed in percentage of control.

### Cellular internalization of NPs

To explore the uptake of NPs by ECs, confocal microscopy and flow cytometry analysis were performed. For confocal microscopy, 1 × 10^5^ cells/well were seeded in a 2-well Lab-Tek Chamber slide (Thermo Scientific, USA). Cells were then treated with or without fluorescent tagged NPs (Fluospheres) purchased from Life Technologies Corporation (Eugene, Oregon) in a concentration of 10 µg/mL alone or with ENA 1 µM for 24 h. After the incubation, cells were washed with PBS and fixed with 4% paraformaldehyde for 15 min followed by counterstaining with DAPI (10 µM). The images were captured using ZEISS LSN980 confocal microscope.

For flow cytometry, cells were treated with or without fluorescent tagged NPs (10 µg/mL) alone and with ENA (1 µM) for 24 h. After treatment, cells were rinsed with PBS, trypsinized, and centrifuged at 1200 rpm. Cells were then fixed with 4% paraformaldehyde for 15 min followed by washing and subsequent resuspension in 200 µL PBS. Fluorescence-activated cell sorting analysis was performed to determine cellular internalization using Beckman Coulter (Cytoflex) instrument and data were analyzed with CytExpert 2.4. The fold change of fluorescence was normalized against the mean fluorescence intensity of the NPs as a reference.

### Western blot analysis

A total of 15 µg per well of proteins were boiled with 1 × loading buffer (Thermo Fisher Scientific, Waltham, MA, USA, cat. no. 39000) for 10 min. Proteins were loaded onto 10% sodium dodecyl sulfate–polyacrylamide gels and separated by electrophoresis, then transferred to polyvinylidene difluoride membranes. After that membranes were blocked with 5% bovine serum albumin (BSA) for 1 h and incubated overnight at 4 °C with primary antibodies against β‐actin (1:20,000; Santa Cruz Biotechnology, Inc), SGLT1 (1:1000; Alomone labs), SGLT2 (1:1000; Alomone labs), p53 (1:1000; Santa Cruz Biotechnology, Inc), p21 (1:1000; Santa Cruz Biotechnology, Inc), Nox2 (1:1000; Proteintech), p22^phox^ (1:1000; Santa Cruz Biotechnology, Inc), eNOS (1:1000; BD Biosciences) diluted in 5% BSA. After washing, membranes were incubated with relevant secondary antibody [horseradish peroxidase (HRP)‐conjugated anti-mouse or anti-rabbit immunoglobulin G (1: 20,000; Cell Signaling Technology)]. Finally, the membranes were washed and treated with enhanced chemiluminescence substrate (GE Healthcare, Little Chalfont, UK, cat. no. RPN2232) and visualized with a chemiluminescence system (UVItec, Cambridge, UK). For densitometric analysis of each blot, Image J software was used, and expression levels of each protein were determined in three independent experiments.

### Measurement of ROS level

A fluorometric microplate assay was used to detect oxidative stress by using two redox-sensitive dyes, dihydroethidium (DHE) and 2′,7′-dichlorodihydrofluorescein diacetate (DCF-DA). In black 96‐well flat‐bottomed plates, PCAECs were seeded at a concentration of 1 × 10^4^ cells/well and allowed to adhere overnight. After washing with PBS, cells were treated with or without NPs (10 µg/mL) alone or with ENA (0.01, 0.1, 1 µM) and further incubated for 24 h. The fluorescence intensity of the oxidation product was measured at excitation/emission wavelengths of 485/535 nm to quantify ROS formation. Intracellular ROS were also determined by fluorescence microscopy. To determine the intracellular ROS levels in PCA, isolated PCA treated with the indicated conditions was embedded in a mould with OCT medium and frozen in liquid nitrogen. Thereafter, cryosections of 10 µm were then subjected to stain with DHE (10 µM) and incubated at 37 °C for 45 min. After all, it is rinsed with 1X PBS and analyzed under a fluorescence microscope.

### Vascular reactivity study

After the dissection of the porcine heart, the left anterior descending coronary artery was excised and cut into 2–3 mm size rings. PCA rings were treated with or without NPs (10 µg/mL) alone or with ENA (0.01, 0.1, 1 µM). PCA rings were suspended in organ baths with oxygenated (95% O_2_ and 5% CO_2_) Krebs bicarbonate solution at 37 °C and measured the changes in isometric tension. Using 80 mM KCl as the maximum contraction, each ring was examined for viability by repetitive contraction after equilibration for 90 min at 5 g tension. After washing for about 30 min, the rings were contracted to about 80% of their maximum contraction using thromboxane mimetic U46619 and a concentration–response curve to bradykinin (BK) was constructed.

### Statistical analysis

Results are presented as the mean ± standard error of the mean (SEM) and were analyzed by one‐way analysis of variance (ANOVA) with a Tukey post-hoc test. For the vascular reactivity study, results were analyzed by two‐way ANOVA with a Bonferroni post-hoc test. All statistical analyses were performed using Prism software (GraphPad Inc., La Jolla, CA, USA). Values of *P* < 0.05 were considered significant.

## Supplementary Information

Supplementary Information.

## Data Availability

The datasets used and/or analyzed during the current study are available from the corresponding authors upon reasonable request.
