# Supplementary material for: SGLT2 inhibition ameliorates nano plastics-induced premature endothelial senescence and dysfunction
Source: Sci Rep. 2023 Apr 17;13:6256. doi: 10.1038/s41598-023-33086-2 (PMC10110533; doi:10.1038/s41598-023-33086-2)

**Supplementary Materials**

**SGLT2 inhibition ameliorates nano plastics-induced premature endothelial senescence and dysfunction**

Bikalpa Dhakal^1,5^, Saugat Shiwakoti^1,5^, Eun-Young Park^1^, Ki-Woon Kang^2^, Valérie B. Schini-Kerth^3^, Sun-Hwa Park^4^, Hye-Young Ji^4^, Joon Seok Park^4^, Ju-Young Ko^1,6^ and Min-Ho Oak^1,6^

^1^College of Pharmacy, Mokpo National University 1666 Yeongsan-Ro, Cheonggye-Myeon, Muan-Gun, Jeonnam 58554, Republic of Korea

^2^Division of Cardiology, Cardiovascular and Arrhythmia Center, Chung-Ang University, Republic of Korea.

^3^Regenerative Nanomedicine, Faculty of Pharmacy, UMR 1260 INSERM, University of Strasbourg, France.

^4^Life Science Institute, Daewoong Pharmaceutical, Yongin, Gyeonggido 17028, Korea.

^5^ These authors contributed equally to this work: Bikalpa Dhakal, Saugat Shiwakoti

^6^ These authors jointly supervised this work: Ju-Young Ko, Min-Ho Oak. Email: [herolegend@mokpo.ac.kr](mailto:herolegend@mokpo.ac.kr); [mhoak@mokpo.ac.kr](mailto:mhoak@mokpo.ac.kr)

**Figure S1. Characterization of NPs. a** FE-SEM imaging of NPs. **b** Particle size distribution. **c** Zeta potential distribution.


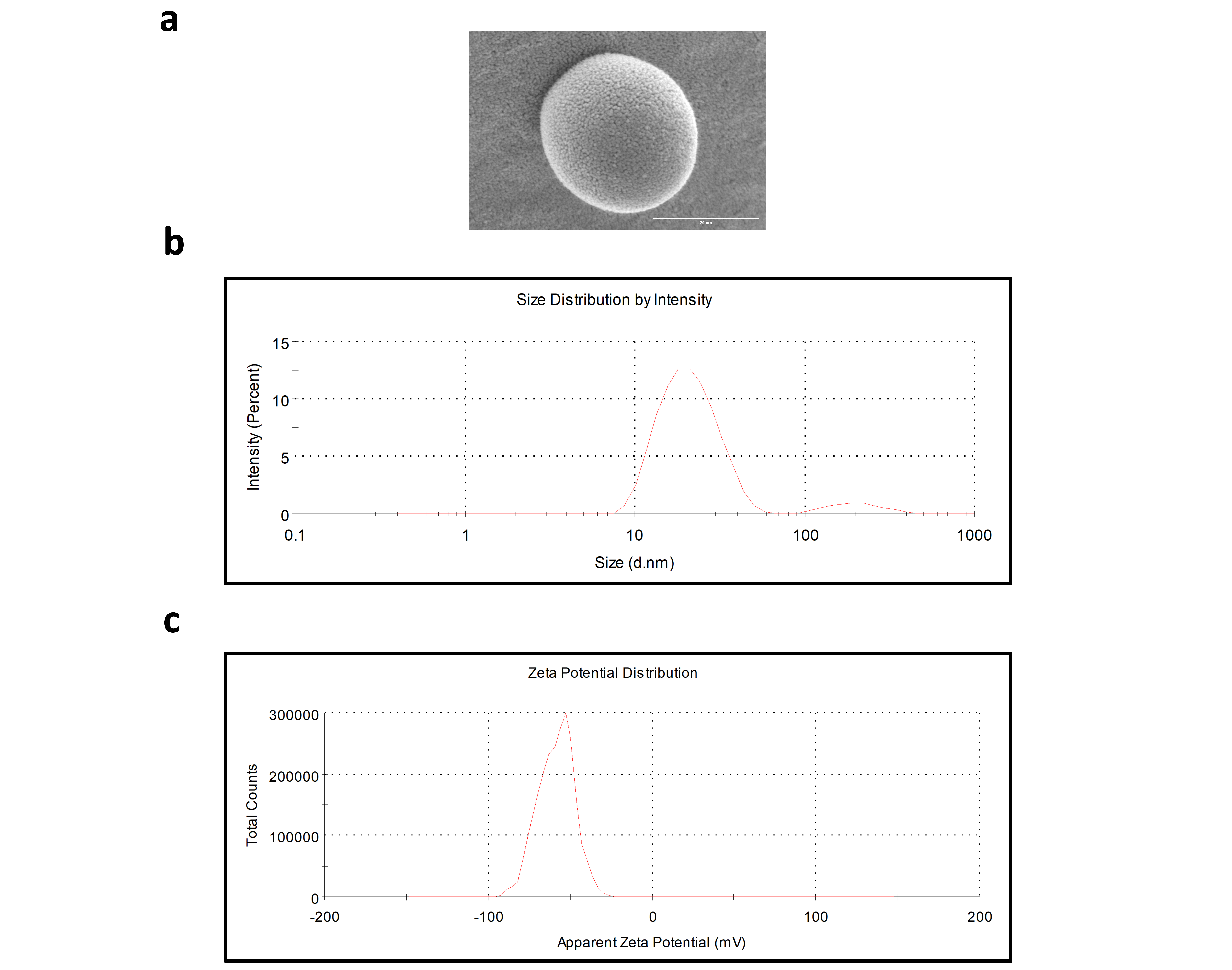


**Fig S2. Images of original uncropped gel of SGLT1 and SGLT2 with corresponding loading control.**


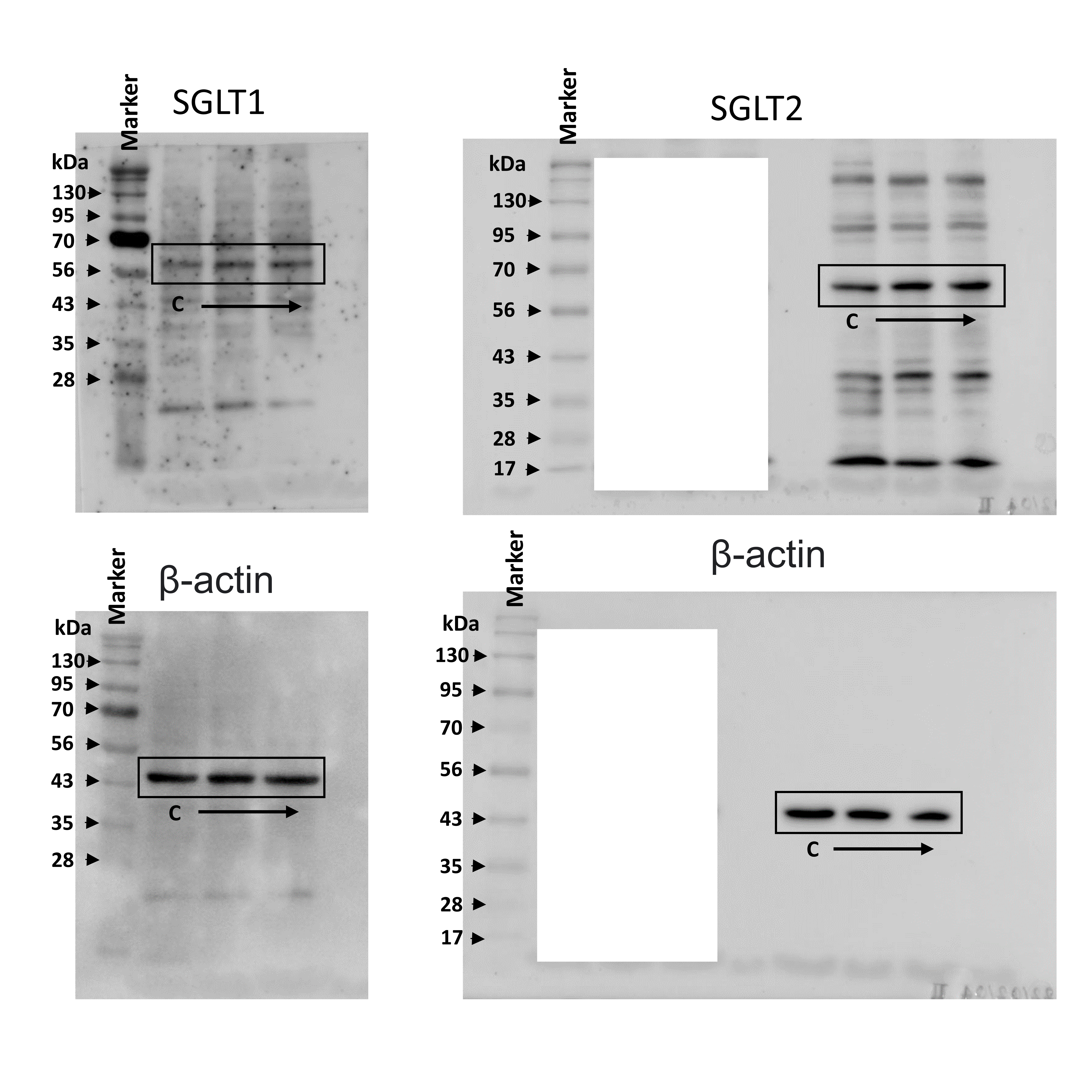


**Fig S3. Images of original uncropped gel of p53 and p21 with corresponding loading control.**


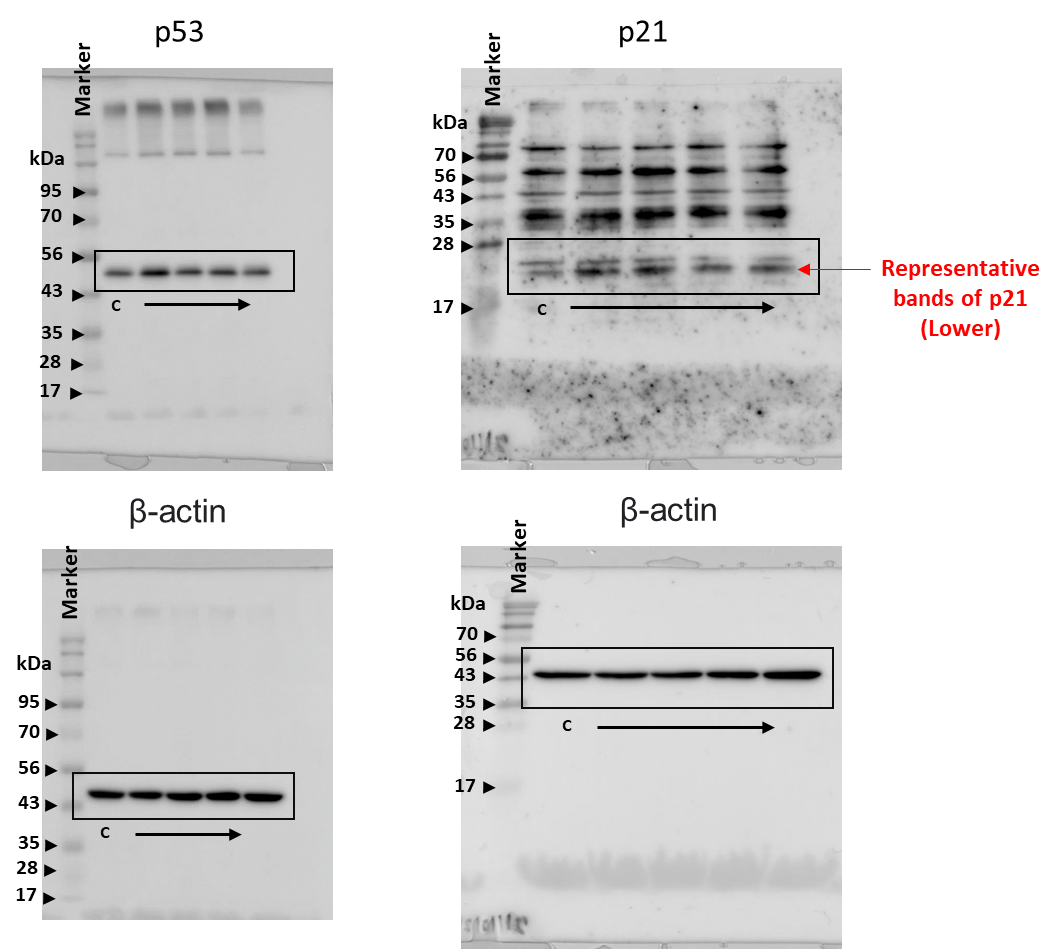


**Fig S4. Images of original uncropped gel of Nox2 and p22^phox^ with corresponding loading control.**


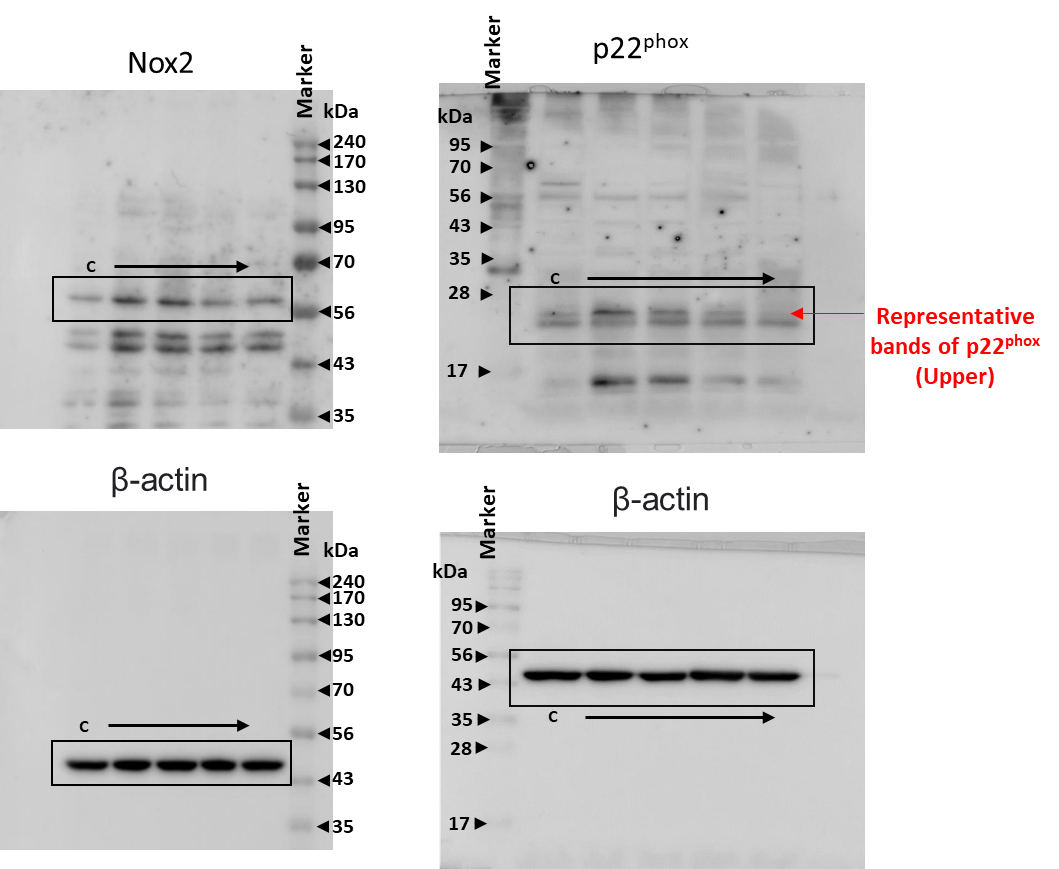


**Fig S5. Images of original uncropped gel of eNOS with corresponding loading control.**


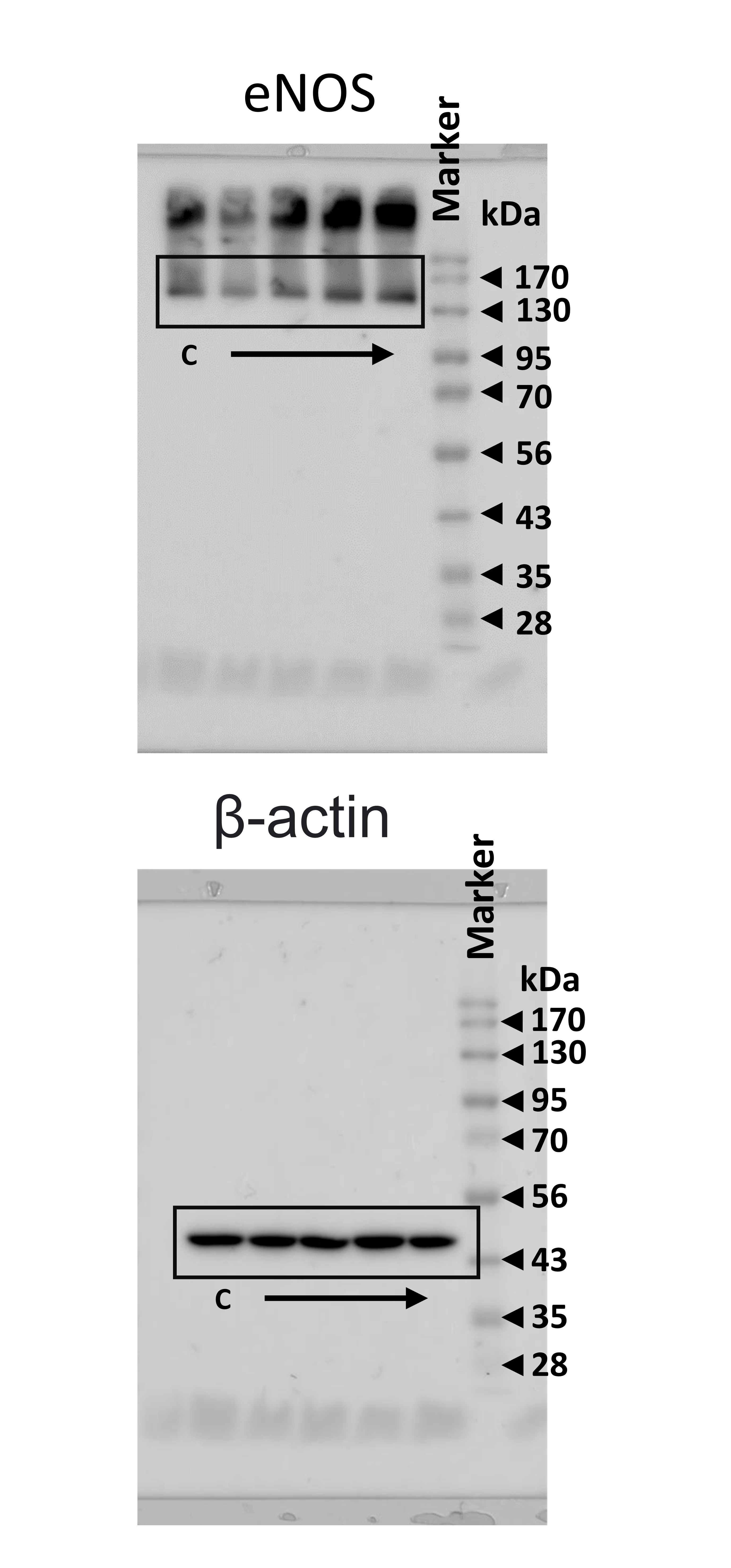

Supplement: Supplementary file 1 — Supplementary Information. [file 41598_2023_33086_MOESM1_ESM.docx]
